# Preclinical development of mesothelin-targeting CAR T cells for the treatment of cholangiocarcinoma

**DOI:** 10.1097/HC9.0000000000000888

**Published:** 2026-01-16

**Authors:** Jinchutha Duangdara, Shaoli Lin, Jessica Hong, Dan Li, Chinnawut Suriyonplengsaeng, Noppadol Larbcharoensub, Kanokpan Wongprasert, Mitchell Ho

**Affiliations:** 1 Department of Anatomy, Faculty of Science, Mahidol University, Bangkok, Thailand; 2 Laboratory of Molecular Biology, Center for Cancer Research, National Cancer Institute, National Institutes of Health, Bethesda, Maryland, USA; 3 Department of Pathology, Faculty of Medicine Ramathibodi Hospital, Mahidol University, Bangkok, Thailand

**Keywords:** bioluminescence tumor imaging, cell immunotherapy, epitope mapping, humanized rabbit monoclonal antibody, hYP218 antibody, liver cancer, xenograft mouse model

## Abstract

**Background:**

Chimeric antigen receptor (CAR) T cell therapy has shown promise in treating hematological malignancies. However, the complex tumor microenvironment of cholangiocarcinoma (CCA) poses significant challenges, particularly due to the lack of clinically validated targets and the presence of fibrosis, which hinders T cell infiltration into tumor sites. Mesothelin (MSLN) is highly expressed in CCA. In this study, we aimed to develop CAR T therapy based on a panel of humanized rabbit monoclonal antibodies targeting various epitopes of MSLN, ranging from the N- to the C-terminus, for CCA.

**Methods:**

MSLN expression was assessed in CCA tissue samples obtained from Thai patients. CAR T cells were generated using various single-chain variable fragment (scFv) constructs, engineered in either VH-linker-VL or VL-linker-VH orientation, targeting non-overlapping epitopes of membrane-bound MSLN: hYP218 (proximal region), hYP223 and hYP3 (middle region), and hYP158 (distal region). The cytotoxicity of MSLN-specific CAR T cells was evaluated in 3 preclinical CCA mouse models: Mz-ChA-1, KMCH, and KMBC.

**Results:**

MSLN was strongly expressed in 79% of the CCA specimens. Among the CAR constructs, hYP218-based CAR T cells—with the VL-linker-VH orientation and CD28-derived hinge and transmembrane domains (CD28HTM)—completely eradicated CCA tumors in all 3 CCA xenograft mouse models (Mz-ChA-1, KMCH, and KMBC). Furthermore, hYP218 VLVH CD28HTM CAR T cells showed strong persistence in mice, with low PD-1 expression (a marker of T cell exhaustion) and minimal adverse effects.

**Conclusions:**

Our findings suggest that MSLN is a promising target for CAR T cell therapy in CCA. CAR T cells engineered with hYP218 VLVH CD28HTM, which targets the membrane-proximal epitope of MSLN, may represent a novel therapeutic strategy for the clinical treatment of CCA.

## INTRODUCTION

Cholangiocarcinoma (CCA) is a bile duct cancer that contributes substantially to cancer-related mortality worldwide.^1^ Although rare in most countries, with annual rates below 6 per 100,000 people, northeastern Thailand reports one of the highest global incidences, approaching 85 per 100,000.^2^ This striking prevalence is largely attributable to infection with the liver fluke *Opisthorchis viverrini*, acquired through eating raw freshwater fish. Chronic infestation of the bile ducts promotes persistent inflammation and carcinogenesis. Most patients present with advanced, unresectable disease, and the current standard treatment with gemcitabine and cisplatin offers only limited survival benefit, highlighting the urgent need for more effective therapies.^1^

Adoptive immunotherapy using chimeric antigen receptor (CAR) T cells has shown remarkable success in hematologic malignancies,^3^ but its use in solid tumors is limited in part by the lack of tumor-specific antigens.^4^ The cell surface glycoprotein mesothelin (MSLN), largely absent from healthy tissues, is a promising therapeutic target.^5^^,^^6^ Preclinical studies demonstrate that MSLN-targeted CAR T cells effectively suppress ovarian, pancreatic, and mesothelioma tumors.^7^ Early-phase clinical trials are investigating MSLN-specific CAR T therapy in multiple solid tumors, including ovarian cancer, mesothelioma, pancreatic cancer, triple-negative breast cancer, gastric cancer, lung cancer, and colorectal cancer (NCT02580747 and NCT03638206).^4^^,^^8^ We and others show that MSLN is highly expressed in CCA, making it an attractive target for antibody-based immunotoxins, though short half-life and immune neutralization limit efficacy.^4^^,^^9^^,^^10^ Therefore, CAR T cells targeting MSLN represent a promising therapeutic strategy for CCA.

The precursor form of MSLN is a ~71 kDa membrane-anchored protein that is processed to a ~40 kDa mature form, which remains attached to the plasma membrane via a glycosylphosphatidylinositol (GPI) anchor after cleavage and shedding of megakaryocyte potentiating factor (MPF), a ~31 kDa soluble protein released into the bloodstream.^6^^,^^11^ Mature membrane-bound MSLN can bind MUC16/CA125, facilitating cell adhesion during tumor progression.^12^^,^^13^ While its normal biological function remains unclear,^14^ MSLN is implicated in tumor progression and CCA development.^10^^,^^15^ Moreover, higher mature MSLN levels correlate with CCA progression in a rat model, supporting its use as a tumor biomarker.^16^

Previously, we developed humanized rabbit monoclonal antibodies targeting non-overlapping epitopes of MSLN.^17^ The antibody hYP158 binds MSLN region I (membrane-distal, residues 296–390), which interacts with MUC16/CA125 and contributes to cancer progression.^13^ Antibodies hYP223 and hYP3 target region II (membrane-middle, residues 391–486), linking the distal and proximal regions, while hYP218 targets region III (membrane-proximal, residues 487–598), anchored via a GPI anchor.^6^^,^^17^ Preclinical studies show that CAR T cells targeting MSLN region III (hYP218 CAR T cells) have superior anti-tumor activity compared with region I-targeted CAR T cells,^7^ exhibiting enhanced persistence, tumor infiltration, and effective elimination of MSLN-positive tumors. These findings indicate that targeting different MSLN regions can yield differential CAR T cell efficacy.

The orientations of VH (heavy chain variable domains) and VL (light chain variable domains) chains in the single-chain variable fragment (scFv) domain can markedly affect antigen binding and tonic signaling, both critical for CAR T cell efficacy.^18^^,^^19^ Optimizing scFv orientation is therefore necessary for each CAR construct to maximize therapeutic activity. In this study, we examined MSLN expression in OV-associated CCA from Thai patients and evaluated the preclinical efficacy of CAR T cells targeting distinct MSLN epitopes with different scFv orientations.

We demonstrated that MSLN was significantly upregulated in OV-associated Thai CCA tissue. Among various MSLN-specific CAR T cells, those with hYP218 VLVH with CD28 hinge and transmembrane (HTM) domain—targeting the membrane-proximal MSLN region—exhibited the most potent anti-tumor effects. This preclinical study introduces the most effective MSLN-specific CAR T cell model for CCA treatment and could provide a promising therapeutic approach for clinical investigations in patients with CCA.

## METHODS

### Cell lines and tissue samples

Human CCA lines (Mz-ChA-1,^20^ KMBC,^21^ and KMCH^22^) were kindly provided by Dr Gregory J. Gores (Mayo Clinic, Rochester, MN, USA). The cell lines were maintained in DMEM or RPMI medium supplemented with 10% fetal bovine serum, 100 U/mL penicillin, 0.1 mg/mL streptomycin, and 2 mmol/L L-glutamine at 37 °C in 5% CO_2_. Intrahepatic CCA tissue samples from 43 Thai patients with OV-associated CCA were obtained via the Departments of Surgery and Pathology, Ramathibodi Hospital, Mahidol University, Thailand, following a previously established protocol^23^. All human research procedures were conducted in accordance with the Declaration of Helsinki and the Declaration of Istanbul and approved by the Ethics Committee of the Faculty of Medicine, Ramathibodi Hospital, Mahidol University (protocol no. 12-58-41). Written informed consent was obtained from all participants before enrollment. Archived and resection specimens were formalin-fixed, paraffin-embedded (FFPE), sectioned at 5 μm, and stained with hematoxylin and eosin (H&E). Two medical pathologists (Chinnawut Suriyonplengsaeng and Noppadol Larbcharoensub) performed histological identification of CCA tissues, and representative areas of the CCA glands were selected for tissue microarray (TMA) generation as previously described.^24^

### Immunohistochemistry analysis of MSLN expression

Immunohistochemistry (IHC) was performed on 43 intrahepatic CCA tissues using the Leica Bond-Max automated system (Leica, Bannockburn, IL, USA). TMA sections underwent deparaffinization, hydration, antigen retrieval, and incubation with anti-MSLN primary antibody (Cat#99966, Cell Signaling Technology, Beverly, MA, USA), followed by horseradish peroxidase (HRP)-conjugated secondary antibody (Cat#7074, Cell Signaling Technology) and visualization with 3,3′-diaminobenzidine (DAB) chromogenic agent (Cat#ab64238, Abcam, Cambridge, UK). Hematoxylin was used for nuclear counterstaining. Ovarian cancer tissue served as a positive control, and an isotype-matched antibody served as a negative control. Two pathologists, blinded to the patients’ clinical data, scored sections using the histology (H)-score method.^25^ The H-score (0–300) was calculated as:


$$\begin{array}{l} H-\mathrm{score}=\left(\mathrm{percentage}\;\mathrm{of}\;\mathrm{cells}\;\mathrm{with}\;\mathrm{weak}\;\mathrm{staining}\times 1\right)\\ +\left(\mathrm{percentage}\;\mathrm{of}\;\mathrm{cells}\;\mathrm{with}\;\mathrm{moderate}\;\mathrm{staining}\times 2\right)\\ +\left(\mathrm{percentage}\;\mathrm{of}\;\mathrm{cells}\;\mathrm{with}\;\mathrm{strong}\;\mathrm{staining}\times 3\right). \end{array}$$

Expression levels were defined as <35 (none), 36–69 (weak), 70–149 (moderate), and ≥150 (strong).

### mRNA analysis of MSLN expression

Total mRNA was extracted from 13 paired intrahepatic CCA and matched resection margins using Tri-Reagent (Molecular Research Center, Inc., Cincinnati, OH, USA) and reverse transcribed with the RevertAid First-Strand cDNA Synthesis Kit (Thermo Scientific, Waltham, MA, USA). Quantitative real-time polymerase chain reaction (qRT-PCR) was performed with SYBR Green Master Mix (Bio-Rad Laboratories, Hercules, CA, USA) in 10 μL reactions (1 μL cDNA, 3 μL nuclease-free water, 0.5 μL of 10 μM primers, 5 μL SYBR mix) on a Bio-Rad CFX96 system. Cycling conditions were 95 °C for 3 minutes, followed by 40 cycles of 95 °C for 5 seconds, 59 °C for 30 seconds, and 72 °C for 30 seconds. The threshold cycle (*C*_t_) values for MSLN were normalized to GAPDH, and relative expression was calculated using the 2^−∆∆Ct^ method with CFX Manager v3.1 (Bio-Rad). Primer sequences: MSLN forward 5′-GCCTTGCTTTCCAGAACA TGA-3′, reverse 5′-GACTGAGCGCCTTCAAATCC-3′; GAPDH forward 5′-CTGACTT CAACAGCGACACC-3′, reverse 5′-TGCTGTAGCCAAATTCGTTG-3′.

### Immunoblotting analysis of MSLN protein expression

Whole protein lysates from 8 CCA specimens and 3 CCA cell lines were prepared using the mammalian protein extraction reagent M-PER (Thermo Scientific) with a protease inhibitor cocktail (Santa Cruz Biotechnology, CA, USA). Protein concentrations were quantified using a Pierce BCA Protein Assay Kit (Thermo Scientific). For immunoblotting, 25 μg of protein was separated on 4%–20% SDS-PAGE and transferred to nitrocellulose membranes. Membranes were incubated overnight at 4 °C with anti-MSLN primary antibody, followed by HRP-conjugated secondary antibody for 2 hours at room temperature. Protein expression was visualized using enhanced chemiluminescence (ECL) (Thermo Scientific) and quantified relative to β-actin or GAPDH using ImageJ 1.47 software (National Institutes of Health, Scion Corporation, Bethesda, MD, USA).

### Flow cytometry analysis of surface MSLN expression

Surface MSLN expression in CCA cell lines was analyzed by flow cytometry. Cells were stained with 5 μg/mL anti-MSLN (rabbit YP218) antibody and detected with goat anti-rabbit IgG-PE (Cat#711-116-152; Jackson ImmunoResearch, West Grove, PA, USA) at 1:200. Data were acquired on a SONY ID7000 (Sony Biotechnology, WA, USA) and analyzed with FlowJo v10.4.2 (FlowJo LLC, Ashland, OR, USA). MSLN molecules per cell were quantified using a standard curve generated with QuantiBRITE PE beads (BD Biosciences, San Jose, CA, USA).

### Generation of MSLN-specific CAR T cells

MSLN-specific CAR T cells were generated as previously described.^26^ Briefly, peripheral blood mononuclear cells (PBMCs) from healthy donors were stimulated with Dynabeads Human T-Activator CD3/CD28 (Invitrogen, Carlsbad, CA, USA) at a 2:1 bead-to-T cell ratio for 24 hours in the presence of IL-2, followed by transduction with lentivirus encoding MSLN-specific CARs at a multiplicity of infection (MOI) of 5. Cell counts were monitored every alternate day, with medium replenished every 2–3 days. CAR T cells were used after 7–9 days of expansion. Amino acid sequences of the VH and VL domains of all scFv constructs are provided in Supplemental Figure S1, http://links.lww.com/HC9/C223.

### Analysis of MSLN antibody binding to MSLN peptide fragments by ELISA

ELISA plates were coated with 5 μg/mL human MSLN fragments fused with rabbit Fc, including full-length MSLN and its distinct regions: distal (region I), middle (region II), and proximal (region III), generated in our laboratory.^13^ Rabbit monoclonal antibodies YP158, YP223, and YP218 (5 μg/mL), or rabbit hybridoma cell culture supernatant containing YP3 monoclonal antibody were added to the wells. Bound antibodies were detected using an anti-rabbit light chain-specific HRP-conjugated secondary antibody (Cat#211-032-171, Jackson ImmunoResearch) at 1:5000. The signal was developed using 3,3′,5,5′-Tetramethylbenzidine (TMB) substrate (Thermo Fisher Scientific), stopped with 2 M sulfuric acid, and optical density (O.D.) was measured at 450 nm using a microplate reader (VersaMax, Molecular Devices, Union City, CA, USA).

### Analysis of CAR transduction efficiency and MSLN fragment binding specificity

CAR transduction efficiency was assessed via expression of truncated human epidermal growth factor receptor (hEGFRt) using 1 µg/mL cetuximab or anti-EGFR human monoclonal antibody. Full-length MSLN binding was evaluated with 5 µg/mL recombinant human MSLN-rabbit Fc chimeric protein (Cat#593206; BioLegend, CA, USA), detected with goat anti-human IgG-PE (Cat#109-116-098; Jackson ImmunoResearch) or goat anti-rabbit IgG-PE (Cat#111-116-144; Jackson ImmunoResearch) at 1:200. CAR specificity to MSLN epitopes was determined using 5 µg/mL MSLN peptide fragments (regions I–III) fused to rabbit Fc, produced in our laboratory, with detection by goat anti-rabbit IgG-PE (1:200). Flow cytometry was performed on a SONY ID7000 (Sony Biotechnology), and data analyzed using FlowJo v10.4.2 (FlowJo LLC).

### In vitro anti-tumor activity of MSLN-specific CAR T cells

MSLN-specific CAR T cells and untransduced (mock) T cells were co-cultured with luciferase- and GFP-expressing CCA cell lines (KMCH, KMBC, Mz-ChA-1) at varying effector-to-target (E:T) ratios for 24 hours. Cytotoxicity was measured using the Luciferase Assay System (Promega, Madison, WI, USA) on a SpectraMax Luminometer (Molecular Devices, Sunnyvale, CA, USA). Supernatants from Mz-ChA-1 co-cultures were analyzed for IL-2, TNF-α, and IFN-γ using the LEGENDplex Human CD8/NK Panel (BioLegend) according to the manufacturer’s instructions, and data were processed with LEGENDplex Data Analysis Software (BioLegend).

MSLN-knockout (KO) KMCH and KMBC cell lines were generated using a lentiviral CRISPR/Cas9 system. sgMSLN primers (forward: 5′-CACCGCAACGGCTCGACCC CTGTTG-3′, reverse: 5′-AAACCAACAGGGGTCGAGCCGTTGC-3′) were ligated into Lenti-CRISPR-V2 (Addgene #52961) for lentivirus production. Cells were transduced, stained with hYP218 antibody, and MSLN-negative cells were sorted using a BD FACSAria Fusion instrument (BD Biosciences) and verified by flow cytometry. Both MSLN-KO and wild-type (WT) cells were co-cultured with hYP218 VLVH CD28 hinge and transmembrane domains (HTM) CAR T cells or mock T cells at varying E:T ratios for 24 hours, and cytotoxicity was measured using a luciferase-based assay.

### Animal studies of MSLN-specific CAR T cell treatment

All animal studies were approved by the Institutional Animal Care and Use Committee at the NIH (protocol LMB-059) and conducted in accordance with the NIH Guide for the Care and Use of Laboratory Animals and ARRIVE guidelines. All mice were housed under a standard light cycle and provided with standard food and water. A 7-week-old female NOD scid gamma (NSG) mouse [National Cancer Institute (NCI) Center for Cancer Research (CCR) Animal Resource Program/NCI Biological Testing Branch] was subcutaneously (s.c.) inoculated with 5 million luciferase/GFP-expressing CCA cells (KMCH, KMBC, and Mz-ChA-1) in 1:1 PBS/Matrigel (BD Biosciences). On days 12 or 14, mice received a single dose of 5 million mock or MSLN-specific CAR T cells via the tail vein. Tumor progression was monitored weekly by bioluminescence imaging (Xenogen IVIS Lumina, PerkinElmer) 15 minutes after intraperitoneal (i.p.) injection of 3 mg D-luciferin (PerkinElmer, Boston, MA, USA). Flux signals (photons/second) were analyzed using Living Image software. Tumor volume was measured with calipers and calculated using the following formula: tumor volume=½ (length × width^2^). Mice were euthanized upon signs of illness.

For the KMCH model, T cells were isolated from spleens using a Tumor Dissociation Kit (Miltenyi Biotec, Bergisch Gladbach, Germany) and cultured with 40 ng/mL IL-7, IL-15, and IL-21 (R&D Systems, Minneapolis, MN, USA). Flow cytometry identified CAR T cells in isolated populations using CD3 (Brilliant Violet 785 anti-human CD3, Cat#31732; BioLegend) and hEGFRt (cetuximab). PD-1 expression in MSLN-specific CAR T cells was also evaluated by flow cytometry. Ex vivo cytotoxicity was assessed by co-culturing CAR T cells with KMCH cells for 36 hours using a luciferase assay. For rechallenge, 3 tumor-free mice from the KMBC hYP218 VLVH CD28HTM treatment group were implanted s.c. with 5 million KMBC cells on day 63 post-treatment, and tumor burden was monitored weekly using a caliper.

### Statistical analysis

Statistical analyses were performed using the GraphPad Prism 6 software (GraphPad Software, Inc., San Diego, CA, USA). MSLN protein and mRNA expression levels in paired CCA and adjacent normal margin tissues were analyzed using a paired Student *t* test (2-tailed), whereas comparisons between 2 experimental groups were analyzed using the Student unpaired *t* test (2-tailed). All experiments were conducted in triplicate, and the data are presented as mean±standard error of the mean (SEM). Kaplan–Meier survival analyses for CCA patients based on IHC levels and CCA mouse models following CAR T cell treatment were evaluated using the log-rank (Mantel–Cox) test. Statistical significance was set at *p*<0.05.

## RESULTS

### MSLN expression in CCA tissues and cell lines

MSLN protein and mRNA expression in Thai CCA tissues was examined by immunoblotting and qRT-PCR, revealing significant upregulation in tumor tissues compared with adjacent normal margins (Figures 1A, B). IHC staining indicated that MSLN was predominantly observed in the cytoplasm and plasma membrane of CCA cells, with prominent apical membrane staining. Semi-quantitative H-score analysis of MSLN staining intensity in CCA tissues indicated positive expression in 86.1% of cases, including strong staining in 79.1% (34/43), moderate in 4.7% (2/43), and weak in 2.3% (1/43), while stromal cells, normal bile ducts, and hepatocytes were negative (Figures 2A–F). In addition, survival analysis indicated that patients with strong-to-moderate MSLN staining had a shorter survival time than those with weak or negative staining (Figure 2G).

**FIGURE 1:**
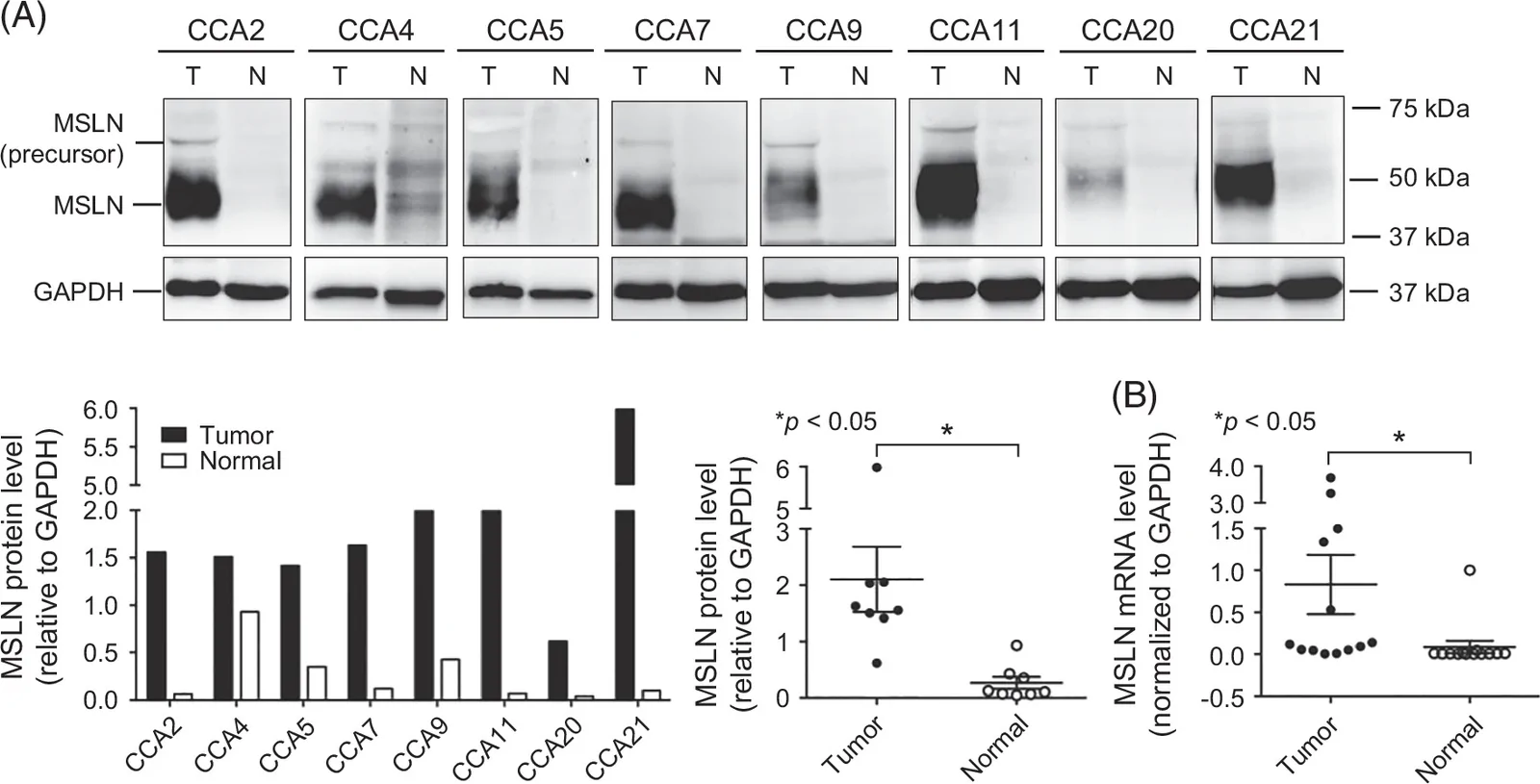
MSLN upregulates in Thai CCA tissues. (A) MSLN protein levels and (B) MSLN mRNA expression were analyzed in paired CCA and adjacent normal margin tissues using immunoblotting (n=8) and qRT-PCR (n=13), respectively. MSLN expression levels were analyzed by normalization to the internal control, GAPDH. Data were analyzed by paired Student *t* test and are represented as means±SEM. Abbreviations: CAA, cholangiocarcinoma; GAPDH, glyceraldehyde 3-phosphate dehydrogenase; MSLN, mesothelin; qRT-PCR, quantitative real-time polymerase chain reaction.

**FIGURE 2:**
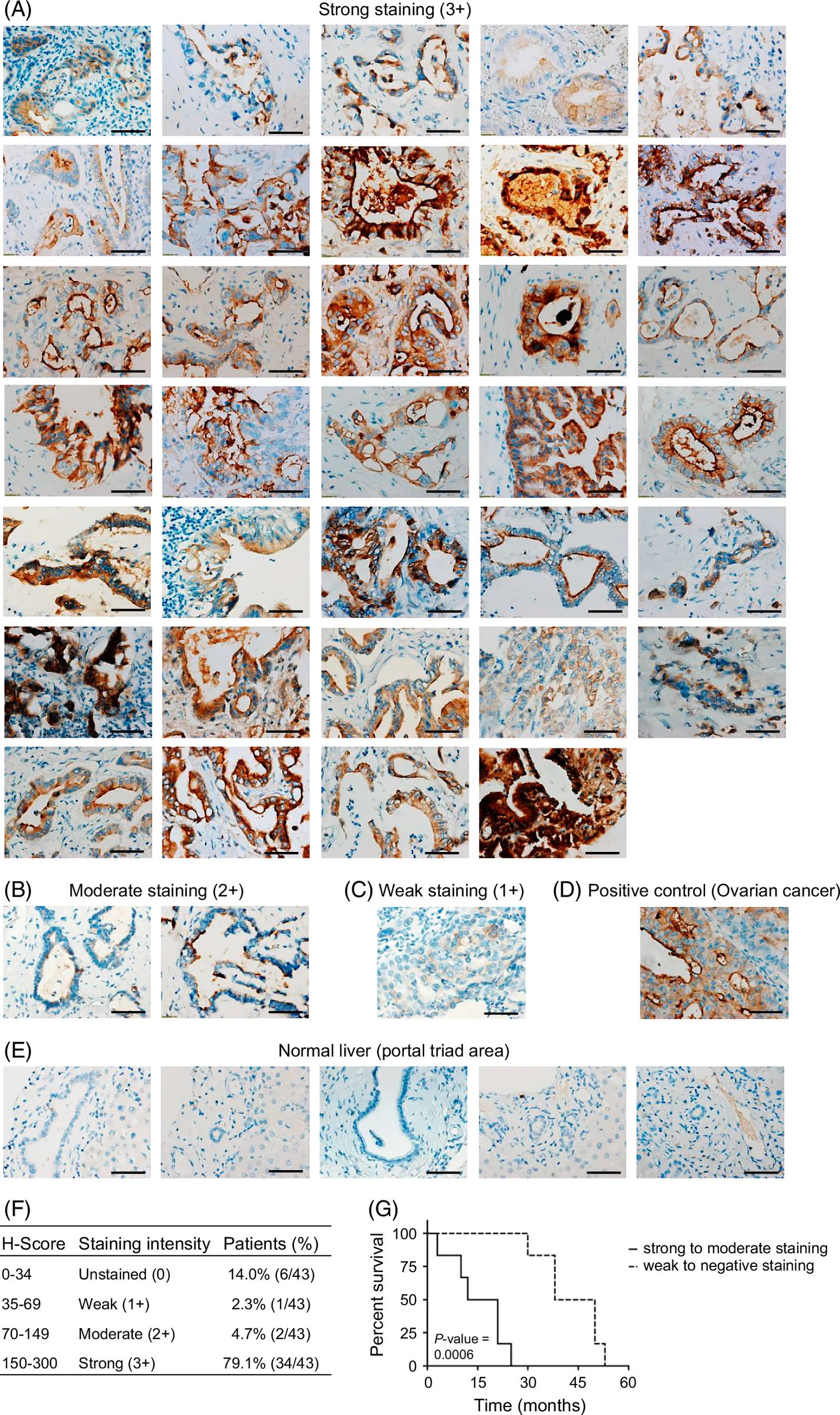
Representative images showing the expression of MSLN in Thai CCA tissues by IHC staining. The IHC staining intensity was evaluated using the H-score. Representative CCA tissues showing (A) strong IHC staining (3+), (B) moderate IHC staining (2+), and (C) weak IHC staining (1+). (D) Ovarian cancer cells were used as positive controls for MSLN staining. (E) Negative staining is observed in cholangiocytes and hepatocytes in normal liver tissues. (F) Percentage of MSLN-positive tumors in Thai CCA patients. (G) Kaplan–Meier survival analysis of patients with CCA based on IHC staining levels. Scale bars=50 μm. Abbreviations: CCA, cholangiocarcinoma; H, histology; MSLN, mesothelin; IHC, immunohistochemistry.

MSLN protein levels in CCA cell lines (KMCH, KMBC, Mz-ChA-1) were assessed by immunoblotting. The result showed high expression of mature MSLN (40 kDa), with Mz-ChA-1 exhibiting the highest levels, while KMCH and KMBC were comparable (Figure 3A). Flow cytometry analysis confirmed surface MSLN expression and quantified molecules per cell using magnetic bead standards, consistent with immunoblot results, with no significant differences among the lines (Figures 3B–D). These findings support MSLN as a potential target antigen for CCA therapy.

**FIGURE 3:**
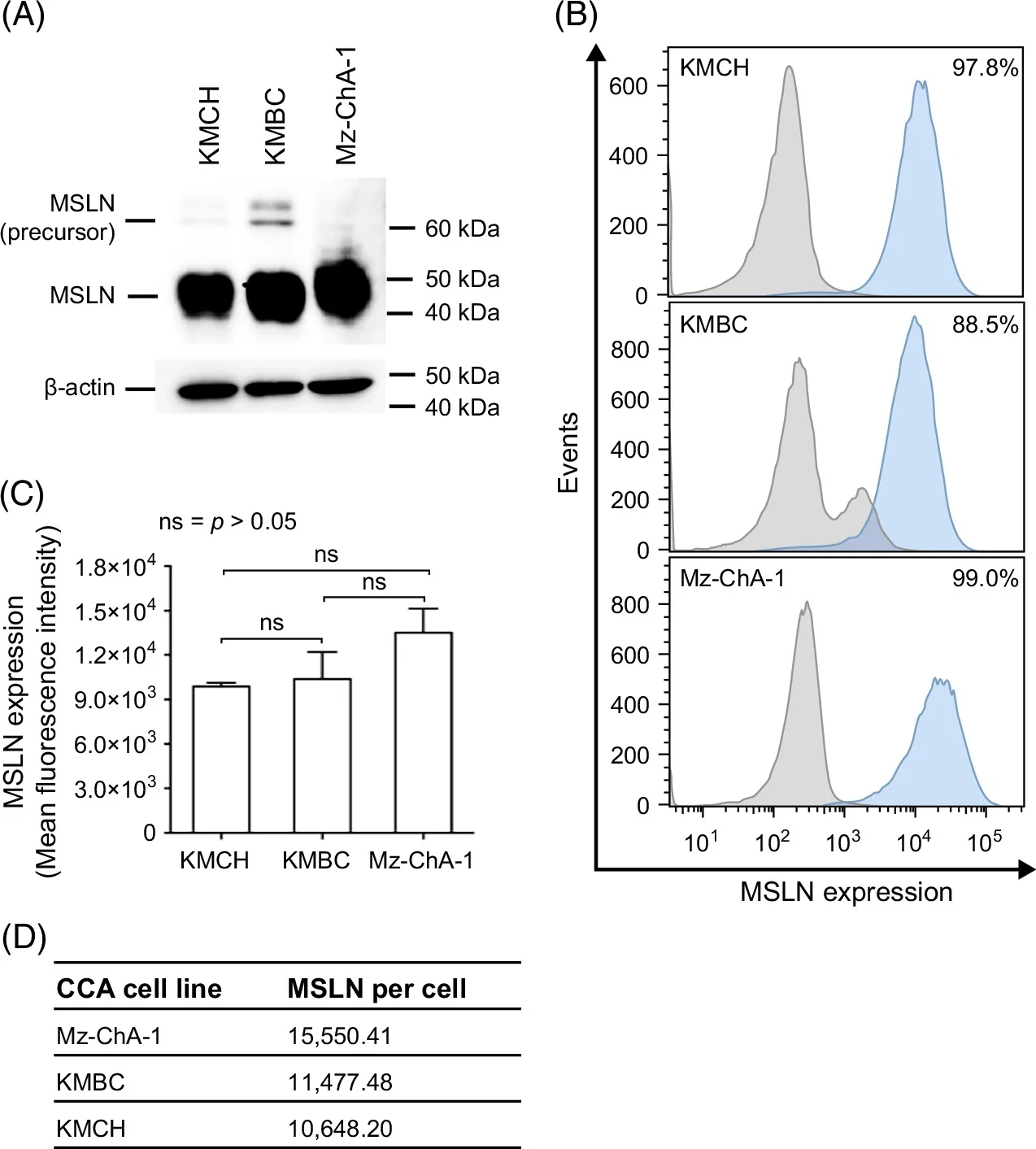
MSLN protein is highly expressed in CCA cell lines. MSLN expression was examined in CCA cell lines, including KMCH, KMBC, and Mz-ChA-1. (A) MSLN expression was examined in total lysates of CCA cell lines by immunoblotting. Β-actin was used as a loading control. (B) Membrane-surface MSLN expression in CCA cell lines was determined by flow cytometry. (C) The representative graph of the fluorescent intensity of MSLN expression on the cell surface is shown. (D) The number of MSLN molecules per cell was quantified using flow cytometry, with a standard curve generated from magnetic beads. Data are represented as means±SEM of triple-independent experiments. Abbreviations: CCA, cholangiocarcinoma; MSLN, mesothelin.

### Development and characterization of MSLN-specific CAR T cells

To develop CAR T cell therapy for CCA, CARs targeting MSLN were constructed using scFv domains in either VH-linker-VL or VL-linker-VH orientation. Our group previously generated rabbit monoclonal antibodies targeting non-overlapping MSLN epitopes: YP158 (membrane-distal, residues 296–390), YP223 and YP3 (membrane-middle, residues 391–486), and YP218 (membrane-proximal, residues 487–581).^17^ Epitope specificity was confirmed by ELISA (Figures 4A, B). Humanized scFvs^27^ were incorporated into CARs containing CD28 hinge and transmembrane domains (HTM), intracellular 4-1BB and CD3ζ signaling domains, and a hEGFRt for transduction monitoring (Figure 4C). hYP218 CAR with a CD8HTM, previously validated in solid tumors, served as a positive control (Figure 4D).

**FIGURE 4:**
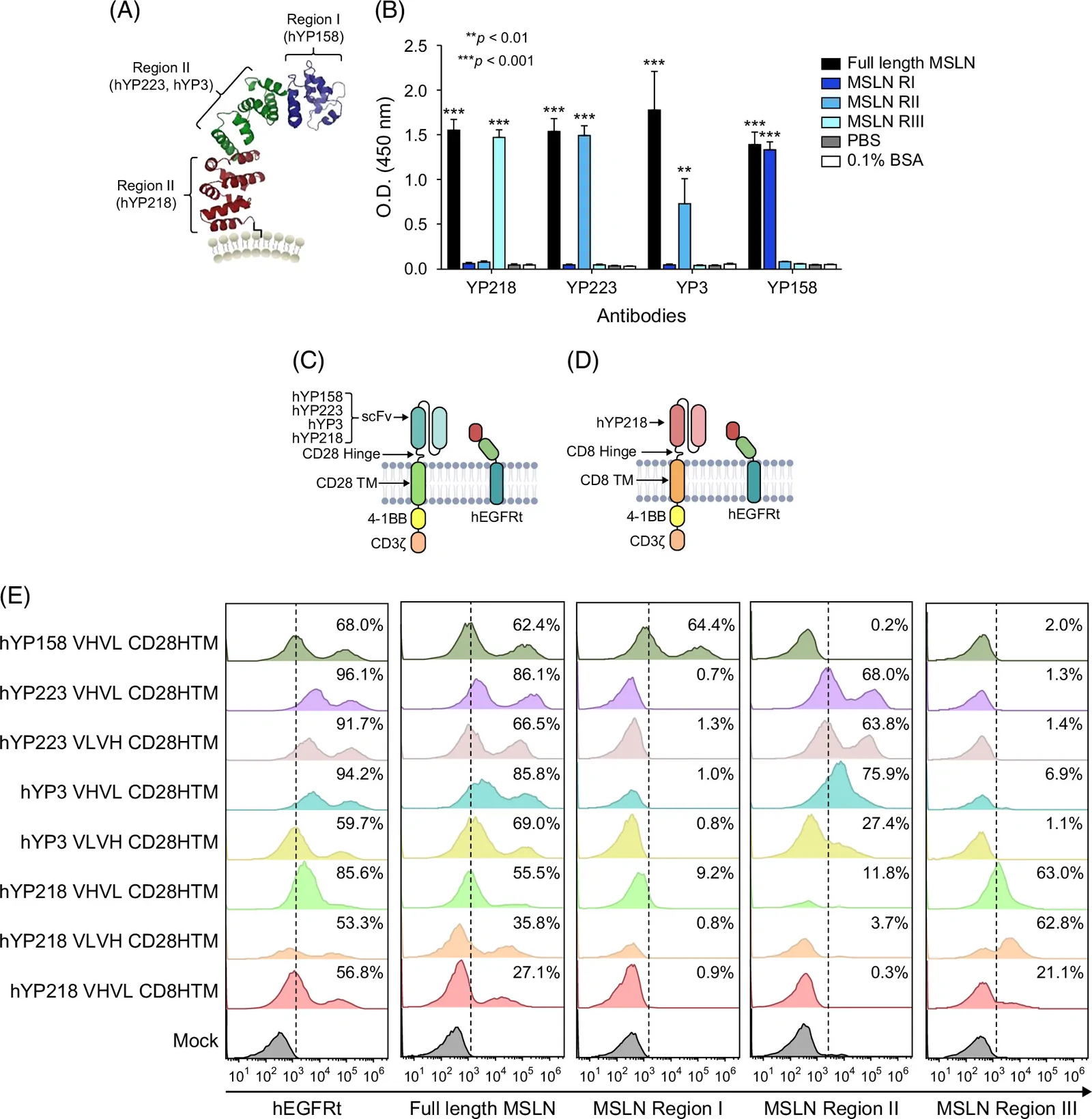
MSLN recognition of anti-MSLN antibodies and the characterization of MSLN-specific CAR T cells. (A) A schematic representation of the anti-rabbit MSLN antibody binding region on non-overlapping epitopes of human MSLN. The model of MSLN protein structure was created by I-TASSER software based on residues 296–598 of the MSLN sequence (image modified from Zhang et al.^17^). (B) The binding specificity of anti-MSLN antibodies, including YP218, YP223, YP3, and YP158, against full-length, distal, middle, and proximal regions of human MSLN proteins was detected by ELISA. (C) and (D) show schematic representations of anti-MSLN CARs containing CD28 hinge and transmembrane domains (HTM) and CD8HTM, respectively (Created in BioRender. Duangdara, J. (2025) https://BioRender.com/lap933z / Mahidol University). (E) The transduction efficiency, MSLN recognition ability, and the binding specificity of CAR T cells to MSLN were evaluated by flow cytometry analysis. The transduction efficiency was determined by the expression of truncated human EGFR (hEGFRt). MSLN recognition ability and the binding specificity were examined by incubating MSLN-specific CAR T cells with full-length, distal, middle, and proximal regions of human MSLN proteins-containing rabbit FC protein fusion, followed by anti-rabbit PE secondary antibody. Data are represented as means±SEM of triple-independent experiments. Abbreviations: CAR, chimeric antigen receptor; hEGFRt, truncated human epidermal growth factor receptor; MSLN, mesothelin; VH, heavy chain variable domains; VL, light chain variable domains.

Flow cytometry analysis was used to assess the transduction efficiency and binding specificity of MSLN-specific CAR T cells. The percentage of hEGFRt-positive cells, indicating successful transduction, ranged from 53% to 96% of the total T cell population. The MSLN recognition ability and specificity of CAR T cells were evaluated by testing their binding to the full-length MSLN protein and its 3 defined fragments (distal, middle, and proximal regions). All MSLN proteins were conjugated to the rabbit Fc segments for detection. Binding analysis revealed that the proportion of CAR T cells recognizing their target MSLN fragments ranged from 27% to 86%. Specifically, 62.4% of hYP158 VHVL CD28HTM CAR T cells were bound to the distal region of the MSLN. In the middle region, over 63% of hYP223 VHVL CD28HTM, hYP223 VLVH CD28HTM, and hYP3 VHVL CD28HTM CAR T cells showed positive binding. However, only 27.4% of the hYP3 VLVH CD28HTM CAR T cells bound to their target epitopes. In the proximal region, more than 62% of the hYP218 VHVL CD28HTM and hYP218 VLVH CD28HTM CAR T cells successfully bound their target fragments. In contrast, the original hYP218 CD8HTM construct demonstrated ~21% binding (Figure 4E). These results demonstrate the successful development of MSLN-specific CAR T cells, characterized by high transduction efficiencies, MSLN recognition capabilities, and region-specific binding properties.

### MSLN-specific CAR T cells kill CCA in vitro

Cytolytic activity of MSLN-specific CAR T cells was assessed by co-culturing 8 constructs (hYP218 VHVL CD8HTM, hYP218 VHVL CD28HTM, hYP218 VLVH CD28HTM, hYP3 VHVL CD28HTM, hYP3 VLVH CD28HTM, hYP223 VHVL CD28HTM, hYP223 VLVH CD28HTM, and hYP158 VHVL CD28HTM) with CCA cell lines (KMCH, KMBC, Mz-ChA-1) for 24 hours, compared with untransduced T cells (mock control). Most constructs efficiently killed target cells, with Mz-ChA-1, the highest MSLN-expressing line, being the most sensitive. Notably, hYP218 VLVH CD28HTM, targeting the proximal MSLN region, exhibited the strongest cytolytic activity across all CCA lines (Figures 5A–C).

**FIGURE 5:**
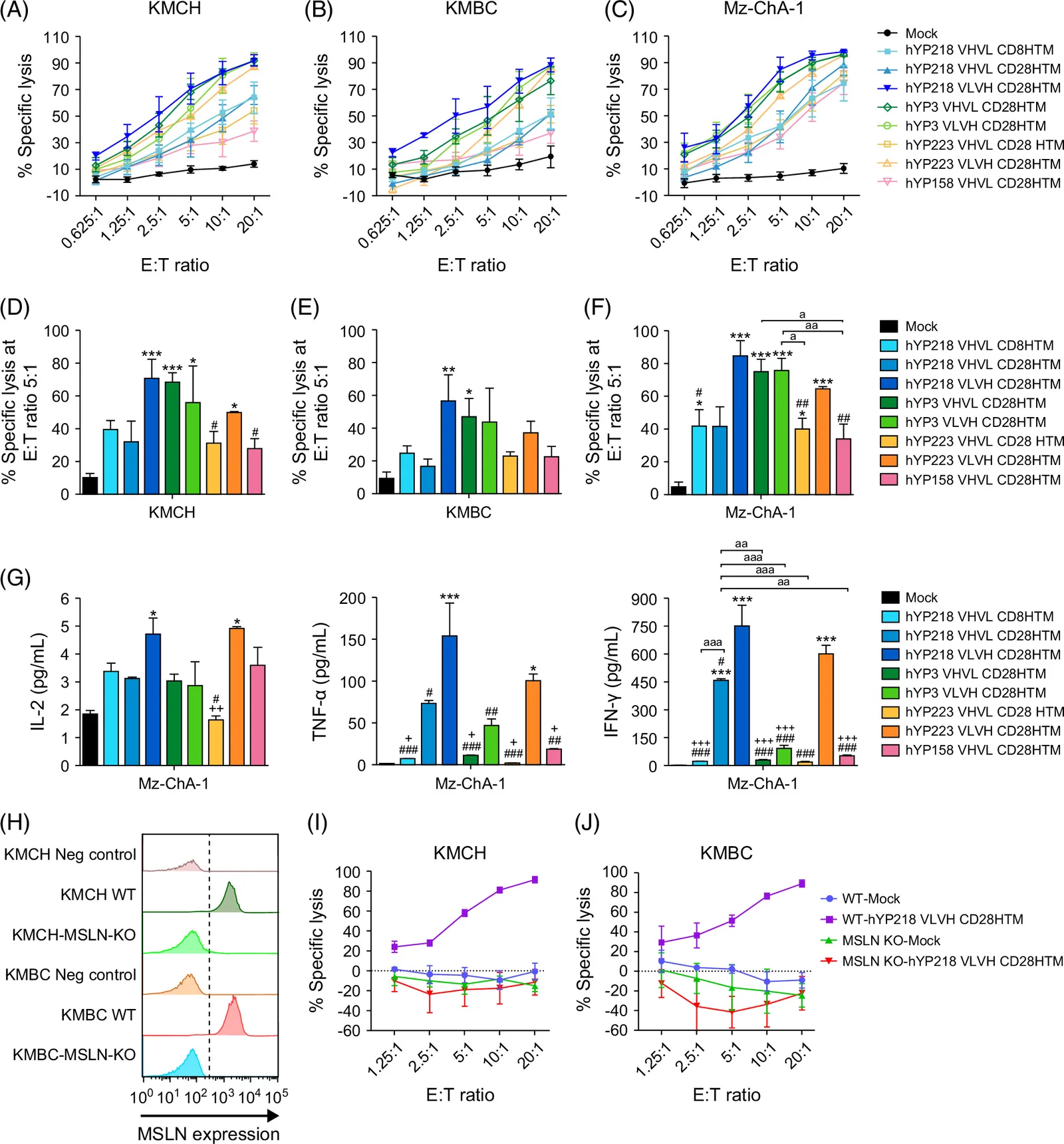
MSLN-specific CAR T cells kill CCA cells in vitro. MSLN-expressing CCA cell lines expressing luciferase protein, including (A) KMCH, (B) KMBC, and (C) Mz-ChA-1, were co-cultured with 8 constructs of MSLN-specific CAR T cells or untransduced (mock) T cells at the indicated effector:target (E:T) ratio for 24 hours. The cytolytic activity was examined using a luciferase-based cytotoxicity assay. The percentage of CCA cell killing, including (D) KMCH, (E) KMBC, and (F) Mz-ChA-1, was measured after co-culture with MSLN-specific CAR T cells at an E:T ratio of 5:1. Pro-inflammatory cytokines, including IL-2, TNF-α, and IFN-γ, were detected in Mz-ChA-1:CAR T cell co-cultured supernatants at an E:T ratio of 5:1 by flow cytometry. (H) Flow cytometry was performed to assess MSLN-knockout (KO) efficiency by detecting MSLN expression. The KMCH-wild-type (WT), KMCH-MSLN-KO, KMBC-WT, and KMBC-MSLN-KO cell lines were stained with the hYP218 antibody followed by a PE-conjugated anti-human secondary antibody. The negative control was stained with the secondary antibody only. MSLN-KO cell lines, including (I) KMCH and (J) KMBC, along with their corresponding WT counterparts, were co-cultured with hYP218 VLVH CD28 CAR T cells or mock T cells at the indicated E:T ratio for 24 hours. Cytolytic activity was then assessed using a luciferase-based cytotoxicity assay. Data are represented as means±SEM from 3 independent experiments. *, **, and *** indicate significant differences from mock at *p*<0.05, *p*<0.01, and *p*<0.001, respectively. #, ##, and ### indicate significant differences from hYP218 VLVH CD28HTM CAR T cells at *p*<0.05, *p*<0.01, and *p*<0.001, respectively. +, ++, and +++ indicate significant differences from hYP223 VLVH CD28HTM CAR T cells at *p*<0.05, *p*<0.01, and *p*<0.001, respectively. a, aa, and aaa indicate significant differences among the groups at *p*<0.05, *p*<0.01, and *p*<0.001, respectively. Abbreviations: CAR, chimeric antigen receptor; CCA, cholangiocarcinoma; E:T, effector-to-target ratios; MSLN, mesothelin.

In Mz-ChA-1 cells, hYP218 VLVH CD28HTM CAR T cells showed the highest cytolytic activity, killing 85.0% of cells, more than twice that of the original hYP218 CD8HTM (41.9%) and VHVL CD28HTM (41.6%) constructs. CAR T cells targeting the middle MSLN region, including hYP3 VLVH CD28HTM, hYP3 VHVL CD28HTM, and hYP223 VLVH CD28HTM, killed 76.1%, 75.4%, and 64.8% of Mz-ChA-1 cells, respectively, whereas hYP223 VHVL CD28HTM was less effective (40.3%). The distal-targeting hYP158 VHVL CD28HTM exhibited minimal cytotoxicity (34.0%) (Figure 5F). A similar hierarchy was observed in KMCH and KMBC cells, with KMCH being more sensitive than KMBC. hYP218 VLVH CD28HTM killed 70.7% of KMCH and 56.5% of KMBC cells. hYP3 VHVL and VLVH CARs killed 68.4% and 55.9% of KMCH cells and 46.9% and 43.8% of KMBC cells, respectively. hYP223 VLVH CD28HTM achieved 49.9% and 37.1% killing in KMCH and KMBC cells, respectively, while other constructs induced <40% cytotoxicity (Figures 5D, E).

To assess CAR T cell functionality, pro-inflammatory cytokines (IL-2, TNF-α, and IFN-γ) were measured in co-culture supernatants of CAR T cells and Mz-ChA-1 cells at an E:T ratio of 5:1 by flow cytometry. All MSLN-specific CAR T cells released significantly higher cytokine levels than mock T cells. Consistent with cytolytic activity, hYP218 VLVH CD28HTM and hYP223 VLVH CD28HTM secreted the highest cytokine levels, while hYP3 constructs showed moderate but significant increases (Figure 5G). These results indicate that MSLN-specific CAR T cells effectively eliminated CCA cells in vitro, with Mz-ChA-1 being the most responsive, and hYP218 VLVH CD28HTM exhibiting the highest cytolytic activity, significantly outperforming the original hYP218 CD8HTM construct.

To further examine the cytotoxic specificity of MSLN-specific CAR T cells, we generated MSLN-KO CCA cell lines, including KMCH and KMBC (Figure 5H). These KO lines, together with their corresponding WT counterparts, were subjected to a cell-killing assay by co-culture with hYP218 VLVH CD28HTM CAR T cells, chosen as a representative construct because it exhibited the highest cytolytic activity among all tested MSLN-specific CAR T cells, or mock T cells at various E:T ratios for 24 hours. The results demonstrated that hYP218 VLVH CD28HTM CAR T cells effectively killed WT cells but not MSLN-KO cells, thereby validating the target specificity of this construct and confirming that the observed CAR T cell-mediated killing is strictly dependent on MSLN expression (Figures 5I, J). Among MSLN-specific CAR T cell constructs, hYP218 VLVH CD28HTM, hYP3 VHVL CD28HTM, and hYP223 VLVH CD28HTM were identified as the most effective and were chosen for subsequent in vivo evaluation.

### MSLN-specific CAR T cells kill CCA in vivo

To evaluate the in vivo anti-tumor efficacy of MSLN-specific CAR T cells, we established s.c. CCA xenograft mouse models. Mz-ChA-1 cells were implanted s.c. in NSG mice, and 2 weeks post-inoculation, a single dose intravenous (i.v.) injection of 5 million CAR T cells was administered. Tumor bioluminescence intensity and tumor volume were monitored weekly (Figure 6A). Five MSLN-specific CAR T cell constructs were tested. Three constructs—hYP218 VLVH CD28HTM, hYP3 VHVL CD28HTM, and hYP223 VLVH CD28HTM—were selected based on their high in vitro tumor lysis activity. Two additional constructs, hYP218 VHVL CD8HTM and hYP218 VHVL CD28HTM, which targeted the proximal region of MSLN, were included for comparison with the most effective clone, hYP218 VLVH CD28HTM. Two weeks after CAR T cell infusion, dramatic tumor regression was observed in all the MSLN-specific CAR T cell treatment groups. Notably, hYP218 VLVH CD28HTM and hYP223 VLVH CD28HTM CAR T cells completely eliminated tumors in all treated mice. In contrast, hYP218 VHVL CD8HTM and hYP218 VHVL CD28HTM CAR T cells required an additional week to achieve similar tumor regression levels. However, tumor bioluminescence reappeared in 2 of the 4 mice in the hYP218 VHVL CD8HTM group. In the hYP3 VHVL CD28HTM group, 2 of 4 mice became tumor-free within 2 weeks, while a slight tumor signal persisted in the remaining mice throughout the experiment (Figures 6B–D). The hYP218 VLVH CD28HTM CAR T cell treatment group achieved a 100% survival rate, whereas only 50% of the mice survived in the hYP223 VLVH CD28HTM group (Figure 6E).

**FIGURE 6:**
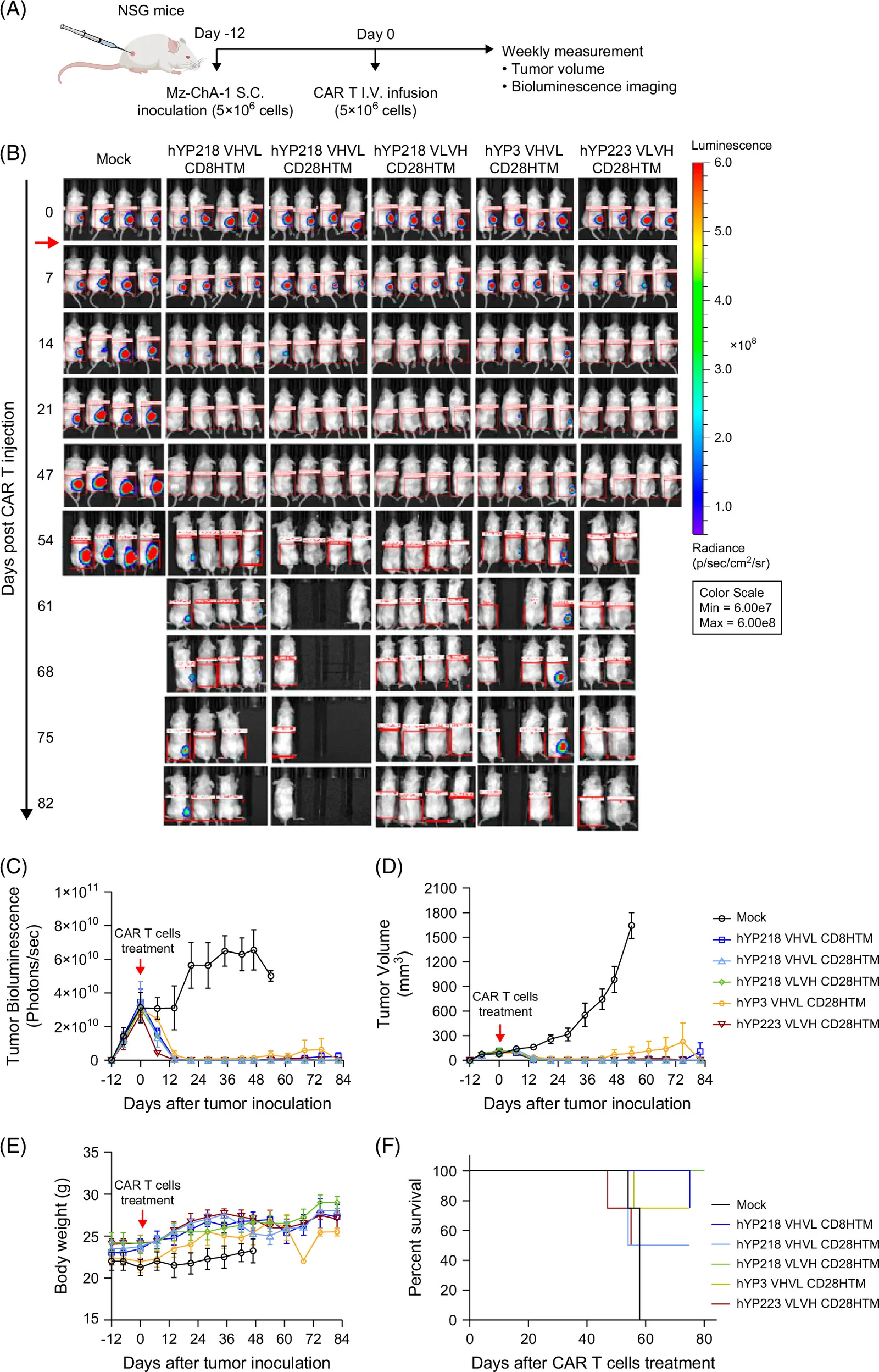
In vivo anti-tumor efficacy of MSLN-specific CAR T cells in Mz-ChA-1 mouse models. (A) Experimental schematic of the Mz-ChA-1 xenograft model (Created in BioRender. Duangdara, J. (2025) https://BioRender.com/m4k3pc2 / Mahidol University). NSG mice were s.c. implanted with 5 million Mz-ChA-1 cells and i.v. infused with 10 million MSLN-specific CAR T cells, including hYP218 VHVL CD8HTM, hYP218 VHVL CD28HTM, hYP218 VLVH CD28HTM, hYP3 VHVL CD28HTM, and hYP223 VLVH CD28HTM, or untransduced (mock) T cells, on day 12. Tumor burden was monitored weekly by bioluminescence imaging and a caliper. (B) Representative bioluminescence image of Mz-ChA-1 tumor growth in mice. (C) The tumor bioluminescence signal is represented as total flux measurements in photons/second. (D) Mz-ChA-1 tumor volume was measured by a caliper. (E) Mouse body weight. (F) Kaplan–Meier survival analysis of Mz-ChA-1 mouse model after CAR T cell treatment. Data are represented as means±SEM. Abbreviations: CAR, chimeric antigen receptor; i.v. intravenous; MSLN, mesothelin; NSG, NOD scid gamma; s.c., subcutaneously.

Given their superior anti-tumor effects in the Mz-ChA-1 model, hYP218 VHVL CD8HTM, hYP218 VLVH CD28HTM, and hYP223 VLVH CD28HTM CAR T cells were further evaluated in a KMCH xenograft model. KMCH cells were implanted s.c., allowed to grow, and treated with a single dose of CAR T cells. The tumor volume and bioluminescence were monitored weekly (Figure 7A). While hYP218 VHVL CD8HTM CAR T cells failed to completely eliminate KMCH tumors, they delayed tumor progression compared with that in the mock group. In contrast, hYP218 VLVH CD28HTM and hYP223 VLVH CD28HTM CAR T cells effectively eradicated tumors in 3 out of 5 mice within 4 weeks. By week 7, all mice treated with hYP218 VLVH CD28HTM CAR T cells were tumor-free, whereas from week 5 onward, the mice in the hYP223 VLVH CD28HTM CAR T cell treatment group were too weak for observation. Therefore, the mice underwent necropsy at week 4 of the treatment (Figures 7B–E). Necropsy revealed systemic inflammation and damage to major organs. To assess CAR T cell persistence, T cells were isolated from the mouse spleens at week 7 (Figure 7F). Ex vivo analysis demonstrated that both hYP218 VHVL CD8HTM and hYP218 VLVH CD28HTM CAR T cells retained significant lytic activity against KMCH cells, with hYP218 VLVH CD28HTM CAR T cells exhibiting a superior killing efficacy (Figure 7G). In addition, the expression of the exhaustion marker PD-1 was significantly lower in hYP218 VLVH CD28HTM CAR T cells than in hYP218 VHVL CD8HTM CAR T cells, indicating improved persistence and reduced exhaustion (Figure 7H).

**FIGURE 7:**
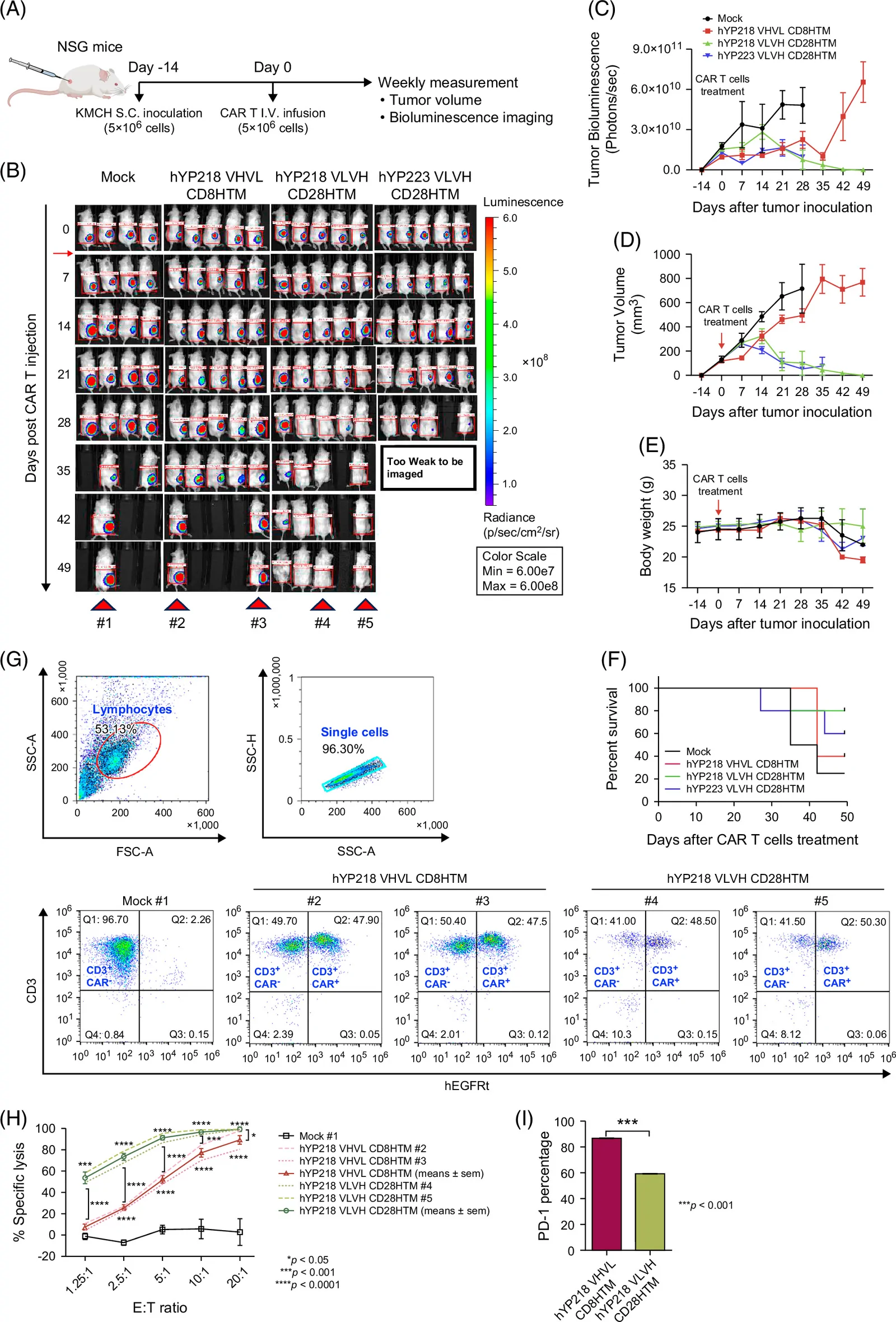
Anti-tumor efficacy of MSLN-specific CAR T cells in KMCH mouse models and ex vivo killing activity. (A) Experimental schematic of the KMCH xenograft model (Created in BioRender. Duangdara, J. (2025) https://BioRender.com/m4k3pc2 / Mahidol University). NSG mice were s.c. implanted with 5 million KMCH cells and i.v. infused with 10 million MSLN-specific CAR T cells, including hYP218 VHVL CD8HTM, hYP218 VLVH CD28HTM, and hYP223 VLVH CD28HTM, or untransduced (mock) T cells, on day 14. Tumor burden was monitored weekly by bioluminescence imaging and a caliper. (B) Representative bioluminescence image of KMCH tumor growth in the mice. (C) The tumor bioluminescence signal is represented as total flux measurements in photons/second. (D) KMCH tumor volume was measured by a caliper. (E) Mouse body weight. (F) Kaplan–Meier survival analysis of the KMCH mouse model after CAR T cell treatment. (G) The identification of CAR T cells, including hYP218 VHVL CD8HTM, hYP218 VLVH CD28HTM, and mock T cells, was performed on T cells isolated from the mice's spleens at week 7 of CAR T cell treatment by flow cytometry. Lymphocytes were gated based on FSC/SSC, singlets were selected, and CD3^+^ T cells were analyzed for hEGFRt expression to identify CAR-expressing cells. (H) The ex vivo killing activity of isolated hYP218 VHVL CD8HTM and hYP218 VLVH CD28HTM T cells, and mock T cells, was assessed against the KMCH cell line at the indicated E:T ratio after 36 hours of co-culture. Cytolytic activity was examined using a luciferase-based cytotoxicity assay. (I) The expression level of the exhaustion marker, PD-1, in the isolated hYP218 VHVL CD8HTM and hYP218 VLVH CD28HTM CAR T cells was determined by flow cytometry. Data are represented as means±SEM. Abbreviations: CAR, chimeric antigen receptor; E:T, effector-to-target ratios; FSC/SSC, forward scatter/side scatter; hEGFRt, truncated human epidermal growth factor receptor; MSLN, mesothelin; NSG, NOD scid gamma; s.c., subcutaneously; VH, heavy chain variable domains; VL, light chain variable domains.

Further evaluation was conducted using the KMBC mouse model to compare the hYP218 VLVH CD28HTM CAR T cells with the original hYP218 VHVL CD8HTM construct. Following s.c. tumor implantation and CAR T cell infusion, the tumor burden was monitored weekly (Figure 8A). We found that hYP218 VLVH CD28HTM CAR T cells completely eradicated KMBC tumors within 3 weeks, whereas hYP218 VHVL CD8HTM CAR T cells failed to regress tumors in mice (Figures 8B–D). To evaluate the long-term efficacy, tumor-free mice treated with hYP218 VLVH CD28HTM CAR T cells were rechallenged with KMBC cells on day 63. The results showed that the resident CAR T cells successfully inhibited tumor growth, demonstrating long-lived, persistent, and sustained anti-tumor potency (Figure 8E).

**FIGURE 8:**
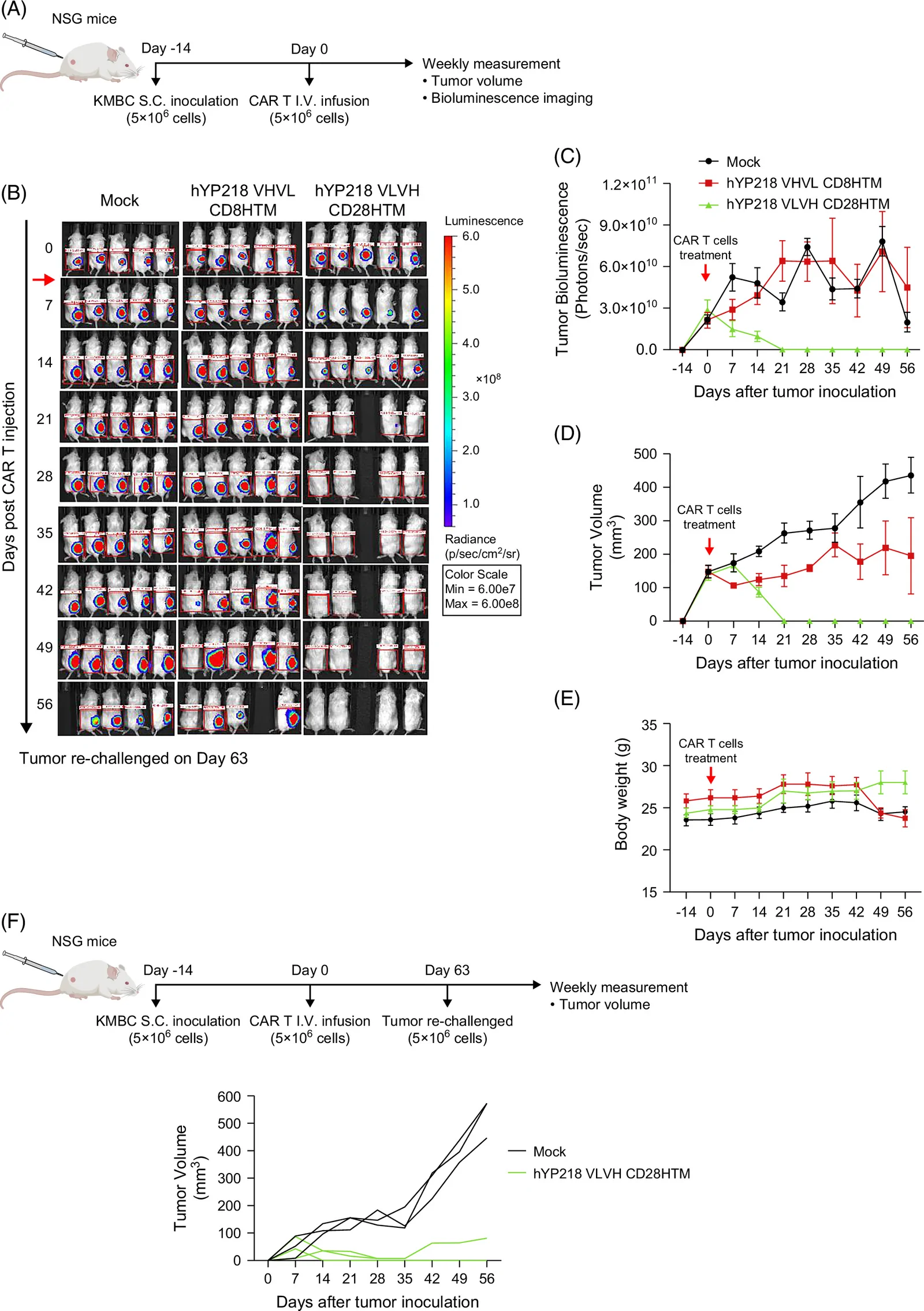
Anti-tumor efficacy of MSLN-specific CAR T cells in KMBC mouse models. (A) Experimental schematic of the KMBC xenograft model (Created in BioRender. Duangdara, J. (2025) https://BioRender.com/m4k3pc2 / Mahidol University). NSG mice were s.c. implanted with 5 million KMBC cells and i.v. infused with 10 million MSLN-specific CAR T cells, including hYP218 VHVL CD8HTM and hYP218 VLVH CD28HTM, and untransduced (mock) T cells, on day 14. Tumor burden was weekly monitored by bioluminescence imaging and a caliper. (B) Representative bioluminescence image of KMBC tumor growth in the mice. (C) The tumor bioluminescence signal is represented as total flux measurements in photons/seconds. (D) The KMBC tumor volume was measured by a caliper. Data are represented as means±SEM. (E) Mouse body weight. (F) KMBC cells were rechallenged s.c. on tumor-free mice on day 63 of hYP218 VLVH CD28HTM CAR T cells treatment, and the tumor size was measured using a caliper (experimental schematic created with BioRender.com). Abbreviations: CAR, chimeric antigen receptor; i.v., intravenous; MSLN, mesothelin; NSG, NOD scid gamma; s.c., subcutaneously.

Taken together, the in vitro and in vivo experiments revealed that among the MSLN-specific CAR T cell constructs evaluated, hYP218 VLVH CD28HTM CAR T cells demonstrated the most robust anti-tumor efficacy against CCA without severe effects on mice. This construct exhibited superior cytolytic activity across all tested CCA cell lines in vitro, accompanied by the highest secretion of key pro-inflammatory cytokines, including IL-2, TNF-α, and IFN-γ. In multiple CCA mouse models, hYP218 VLVH CD28HTM CAR T cells completely eradicated tumors, outperformed the original CD8HTM-based construct in persistence, and maintained potent ex vivo killing activity after 7 weeks. Furthermore, these CAR T cells displayed significantly lower PD-1, indicating reduced exhaustion and enhanced longevity. Importantly, this construct provided durable tumor control, as demonstrated by the successful inhibition of rechallenged tumors while maintaining a high survival rate and minimal systemic toxicity in treated mice. Collectively, these findings highlight hYP218 VLVH CD28HTM CAR T cells as a highly promising and potent therapeutic strategy for CCA treatment.

## DISCUSSION

This study revealed 2 main findings: MSLN was highly expressed in 79% of Thai CCA tissues and correlated with poor clinical outcomes, and CAR T cells with hYP218 Fv (VL with a GS flexible linker followed by VH) plus a CD28 hinge and transmembrane domain (HTM) exhibited greater anti-CCA effects in preclinical models compared with other tested constructs.

Overexpression of MSLN has been reported in multiple solid tumors.^5^^,^^28^ MSLN-specific CAR T cell therapy has demonstrated significant anti-tumor effects in preclinical models^7^ and is currently being evaluated in early-phase clinical trials of various tumors,^4^^,^^10^ supporting its therapeutic potential. While prior studies documented MSLN expression in CCA specimens from American patients,^9^ CCA predominantly affects Asian populations, especially in Northern Thailand, where liver fluke infection is a major risk factor.^1^^,^^2^ Immunohistochemical (IHC) analysis of Thai OV-associated CCA specimens revealed high MSLN expression in 79% (34 of 43) of cases. Consistent with our previous findings, MSLN expression was restricted to CCA cells, with absent expression in adjacent normal cholangiocytes and hepatocytes.^9^ MSLN expression was also high in CCA cell lines, including Mz-ChA-1, KMCH, and KMBC, which were therefore selected as representative models to evaluate MSLN-targeted CAR T cell therapy.

Several factors influence the efficacy of CAR T cell therapy.^29^ To optimize CAR T cell efficacy, we generated constructs targeting non-overlapping MSLN epitopes with either VH-linker-VL or VL-linker-VH orientation.^17^ All constructs showed high transduction efficiency and specificity for their respective MSLN regions. CAR T cells targeting the membrane-proximal epitope of MSLN (hYP218 VLVH CD28HTM) achieved complete tumor regression in murine models, with superior cancer cell elimination, long-term persistence, and robust anti-tumor cytokine production. In contrast, CARs targeting the middle (hYP3 and hYP223) or distal (hYP158) epitopes were less effective. Notably, hYP218 VHVL CD28HTM was inferior to its VLVH counterpart, underscoring the critical influence of scFv orientation on therapeutic performance.

HTM domains also significantly influenced CAR T cell function.^30^^–^^32^ Typically, HTM regions are derived from natural T cell components, such as CD28 or CD8, to minimize unintended immune interactions and enhance clinical safety.^33^ CD28HTM CAR show higher dimerization, increased cytokine production, and activation-induced cell death compared with CD8HTM CAR,^30^ motivating their use to balance efficacy and safety. In addition, the orientation of the scFv affects antigen binding and tonic signaling, which in turn influences CAR T cells' function and exhaustion.^18^^,^^19^ The distance between the third complementarity-determining region (CDR3) of the heavy chain and the scFv linker can influence antigen binding, and studies suggest that VLVH orientation often improves surface expression and target binding compared to VHVL.^34^ In line with this, hYP218 and hYP223 CAR T cells with VLVH orientation were more effective, whereas hYP3 showed no significant difference. These findings highlight scFv orientation as a critical determinant of CAR T cell therapeutic performance.

Among the tested CCA cell lines, Mz-ChA-1 was most sensitive to CAR T therapy, whereas KMBC and KMCH were less responsive. Although MSLN expression was comparable, differences in tumor differentiation, aggressiveness, and intrinsic mutation profiles likely influenced efficacy.^35^^,^^36^ Each line harbors distinct mutations, with KMBC and KMCH displaying more aggressive, poorly differentiated phenotypes compared with the well-differentiated Mz-ChA-1.^20^^–^^22^^,^^37^^,^^38^ These differences are associated with disease progression and immune evasion.^39^^–^^41^ The desmoplastic stroma and immune components of the CCA tumor microenvironment (TME), such as cancer-associated fibroblasts (CAFs) and tumor-associated macrophages (TAMs), modulate treatment response.^42^ Overall, both CAR design and tumor-intrinsic features contribute to therapeutic efficacy.

Although hYP223 VLVH CD28HTM showed strong anti-tumor efficacy, it also caused systemic inflammation and reduced survival, indicating CAR-intrinsic toxicity possibly linked to tonic signaling or cytokine-driven inflammation^43^^,^^44^ rather than on-target/off-tumor effects, as all constructs bound human MSLN without cross-reactivity to murine MSLN (Supplemental Figure S2, http://links.lww.com/HC9/C223).^17^ The use of immunodeficient NSG mice, which lack functional lymphocytes, limits the recapitulation of immune-stromal interactions in the CCA microenvironment, including immunosuppressive stroma and tumor-associated immune cells.^45^ While NSG xenografts remain a standard preclinical platform for evaluating CAR T cell efficacy,^7^ orthotopic CCA animal models with a normal immune system may better reflect the clinical TME and improve translational relevance.^46^ The systemic toxicity of hYP223 CAR T cells led us to exclude it from clinical development. We instead focused on the most effective and well-tolerated construct, hYP218 VLVH. Our present study demonstrates that the hYP218 VLVH CAR construct is safe and well-tolerated in mice. Although in vivo toxicological data were limited, prior studies with hYP218-based CARs or immunotoxins showed potent anti-tumor activity while preserving organ function and normal serum chemistry, supporting a favorable safety profile of CAR T cells.^7^^,^^17^ However, further histological evaluation of MSLN-expressing tissues and cytokine profiling will be needed to confirm CAR T cell safety in vivo.

In conclusion, our findings demonstrate that MSLN is a promising therapeutic target for CCA, and MSLN-specific CAR T cells, particularly those based on hYP218 scFv, show strong preclinical efficacy. Our results further highlight that both epitope selection and scFv orientation are critical determinants of CAR T cell efficacy. Overall, this study suggests that CAR T cells based on hYP218 VLVH CD28HTM have significant potential as a novel therapeutic strategy for the clinical treatment of CCA.

## Supplementary Material
