# Supplementary material for: Preclinical development of mesothelin-targeting CAR T cells for the treatment of cholangiocarcinoma
Source: Hepatol Commun. 2026 Jan 16;10(2):e0888. doi: 10.1097/HC9.0000000000000888 (PMC13671593; doi:10.1097/HC9.0000000000000888)
Supplement: Supplementary file 1 [file hc9-10-e0888-s001.pdf]

Supplementary Figure S1

Amino acid sequences of VH and VL domains of all scFv constructs with CDR regions defined by IMGT numbering

scFv	VH	VL
**hYP218**	EVQLVESGGGLVQPGGSLRLSCAAS**GFDLGFYFYA**CWVRQAPGKGLEWVSC**IYTAGSGST**YYASWAKGRFTISRDNSKNTLYLQMNSLRAEDTAVYYC**ARSTANTRSTYYLNL**WGQGTLVTVSS	DIQMTQSPSSLSASVGDRVTITCQAS**QRISSY**LSWYQQKPGKVPKLLIY**GAS**TLASGVPSRFSGSGSGTDFTLTISSLQPEDVATYYC**QSYAYFDSNNWHA**FGGGTKVEIK
**hYP3**	EVQLVESGGGLVQPGGSLRLSCAASG**IDFSRYY**MCWVRQAPGKGLEGIAC**IYIGGSGST**YYASWAKGRFTISRDNSKNTLYLQMNSLRAEDTAVYYC**ARGTNLNYIFRL**WGQGTLVTVSS	DIQMTQSPSSLSASVGDRVTITCQAS**QSINNG**LAWYQQKPGKVPKLLIY**SAS**NLESGVPSRFSGSGSGTDFTLTISSLQPEDVATYYC**QCIWDGNSYVNA**FGGGTKVEIK
**hYP158**	EVQLVESGGGLVQPGGSLRLSCAAS**GFSFSGDYY**MCWVRQAPGKGLEWIA**CIGGGSNTAT**YYATWAKGRFTISRDNSKNTLYLQMNSLRAEDTAVYYC**ARDLGFVDYALEL**WGQGTLVTVSS	DIQMTQSPSSLSASVGDRVTITCQAS**ENMYNS**LAWYQQKPGKVPKLLIY**RAS**TLESGVPSRFSGSGSGTDFTLTISSLQPEDVATYYC**QCTFYSHNNNYGGA**FGGGTKVEIK
**hYP223**	EVQLVESGGGLVQPGGSLRLSCAAS**GLDFSSSYW**ICWVRQAPGKGLEWIGC**RHTFTANT**WSASWVNGRFTISRDNSKNTLYLQMNSLRAEDTAVYYC**ARDESNNDGWDFKL**WGQGTLVTVSS	DIQMTQSPSSLSASVGDRVTITCQAS**QSISNY**LAWYQQKPGKVPKLLIY**QAS**TLAPGVPSRFSGSGSGTDFTLTISSLQPEDVATYYC**QQGYTSSNVENV**FGGGTKVEIK

CDR1
CDR2
CDR3

scFv linker sequence : GGGGSGGGGSGGGGS

Supplementary Figure S2

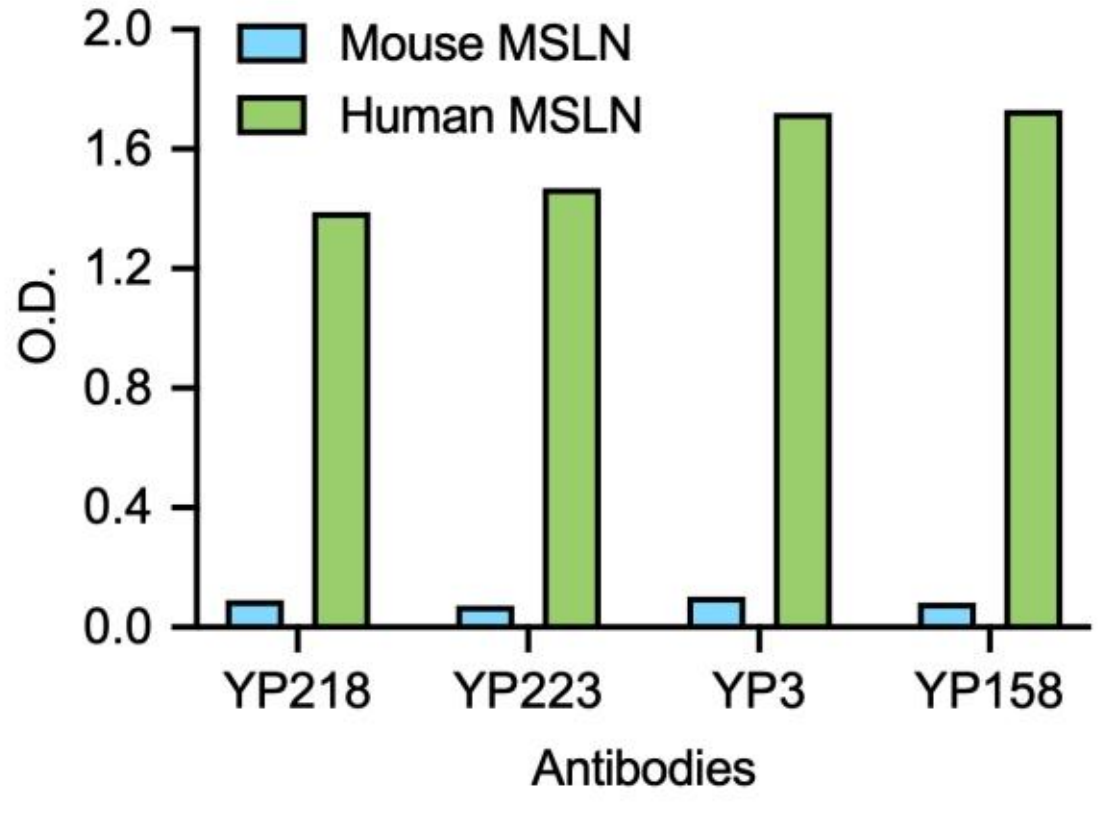

ELISA assay demonstrating the binding specificity of MSLN-targeted CAR constructs to recombinant human and mouse mesothelin proteins
